# Long‐term predialysis blood pressure variability and outcomes in hemodialysis patients

**DOI:** 10.1111/jch.14398

**Published:** 2022-01-28

**Authors:** Jingjuan Yang, Jian Huang, Biying Yu, Qian Zhang, Shanshan Zhang, Longlong Wu, Lin Luo, Lizhu Li, Li Li, Fei Han, En Yin Lai, Yi Yang

**Affiliations:** ^1^ Department of Nephrology the Fourth Affiliated Hospital Zhejiang University School of Medicine Yiwu China; ^2^ Department of Nephrology Central Hospital of Jinhua Jinhua China; ^3^ Hemodialysis Quality Control Center of Jinhua Jinhua China; ^4^ Kidney Disease Center the First Affiliated Hospital Zhejiang University School of Medicine Hangzhou China; ^5^ Department of Physiology School of Basic Medical Sciences Zhejiang University School of Medicine Hangzhou China

**Keywords:** all‐cause mortality, hemodialysis, MACE, predialysis blood pressure

## Abstract

Blood pressure variability (BPV) is significantly associated with cardiovascular diseases (CVD) and mortality in hemodialysis patients. However, the relationship between blood pressure and CVD in hemodialysis patients is complex and affected by many factors. The present study aimed to assess the association of long‐term predialysis BPV with all‐cause mortality and major adverse cardiovascular events (MACE). One thousand seven hundred twenty‐seven patients receiving maintenance hemodialysis were recruited in nine hemodialysis centers. Predialysis BPV was assessed over 1‐year intervals. Outcomes included all‐cause mortality and MACE during follow‐up periods. The mean age of the final cohort was 59 years, of which 57% were males. Greater predialysis systolic BPV was associated with an increased risk of all‐cause mortality (adjusted hazard ratio, 1.101; 95% confidence intervals 1.064–1.140) and MACE (adjusted hazard ratio, 1.091; 95% confidence intervals 1.059–1.125). Results were similar when systolic BPV was stratified by baseline systolic blood pressure. In conclusion, greater predialysis BPV among hemodialysis patients was associated with all‐cause mortality and MACE. Strategies to reduce blood pressure variability might be beneficial for hemodialysis patients.

## 1 INTRODUCTION

Hypertension is reported in greater than 90% of patients receiving long‐term hemodialysis and may lead to an increased morbidity of cardiovascular diseases (CVD).^1^, ^2^, ^3^ Numerous cardiovascular factors were associated with mortality and CVD in hemodialysis patients, including blood pressure (BP) fluctuations and intradialytic hypotension; cardiac structural changes; neurohormonal instability; and autonomic instability.^4^ However, the relationship between BP and CVD in hemodialysis patients is complex and affected by many factors.^5^

Fluctuations in BP or BP variability (BPV) is common in hemodialysis patients. Those fluctuations entail changes in systolic blood pressure (SBP) and diastolic blood pressure (DBP) that occurs before (predialysis), during (intradialytic), or after (postdialysis) the treatments.^6^ BPV can be short‐term, midterm, or long‐term. Short‐term BPV includes beat‐to‐beat, minute‐to‐minute, hour‐to‐hour, and circadian variability over a period of 24 hours; midterm BPV includes variability over a periods of days; long‐term BPV includes variability over weeks, months, seasons, and even years.^7^

In hemodialysis patients, the relationships between short‐term BPV and prognosis have been extensively studied.^8^, ^9^, ^10^, ^11^, ^12^ However, the effects of long‐term BPV, especially variability over years, on prognosis in hemodialysis patients are less investigated. The goal of this study was to elucidate the role of some readily available clinical and demographic factors in BPV, including predialysis BP and BPV in all‐cause mortality and major adverse cardiovascular events (MACE). The present study recruited hemodialysis patients from nine centers to assess the association of long‐term predialysis BPV with all‐cause mortality and MACE.

## 2 METHODS

### 2.1 Study participants and study design

#### 2.1.1 Initial screening stage

Adult patients (aged ≥18 years), who had received maintenance hemodialysis for more than 3 months prior to August 1, 2018, were recruited from the Fourth Affiliated Hospital, Zhejiang University School of Medicine and eight public hospitals in Jinhua city, Zhejiang Province of China (Central Hospital of Jinhua, People's Hospital of Jinhua, Central Hospital of Yiwu, People's Hospital of Yongkang, People's Hospital of Pujiang, Dongyang Hospital of Traditional Chinese Medicine, People's Hospital of Lanxi, and Lanxi Hospital of Traditional Chinese Medicine).

Patients with any one of the following criteria were excluded: (1) died, or underwent kidney transplantation, or switched to peritoneal dialysis, or transferred to a different renal unit between August 1, 2018 and July 31, 2019; (2) had a history of arrhythmia, including atrial fibrillation, atrial flutter, atrial tachycardia, ventricular tachycardia, ventricular fibrillation, and II‐ or III‐degree atrioventricular block; (3) incomplete dialysis records (no data for > 3 months).^13^

#### 2.1.2 Follow‐up stage

After the initial screening, we followed patients from August 1, 2019, to July 31, 2020. Patients were censored from the analyses if they underwent kidney transplantation, switched to peritoneal dialysis, transferred to a different renal unit, or were lost to follow‐up.

This study was approved by the Research Ethics Committee of the Fourth Affiliated Hospital, Zhejiang University School of Medicine (K20190047) and was recorded in the Chinese Clinical Trial Register (ChiCTR2000028945). All methods were performed in accordance with the approved guidelines and relevant regulations.

### 2.2 Study protocol

The hemodynamic data was collected prospectively from all of the hemodialysis sessions of the participants between August 1, 2018, and July 31, 2019. At each session, patients were assessed for pre‐ and post‐ dialysis weight, and predialysis and intradialytic SBP, DBP and heart rate. The BP and heart rate were measured with patient seated in a chair with feet on the floor and back supported. Measurement was made by trained research assistants with a validated automated oscillometric brachial BP monitor (Omron 907XL; Omron Healthcare, Lake Forest, IL, USA). Predialysis BP and heart rate were measured after a 10‐minute rest period in a chair before dialysis. BP was measured three times consecutively before each dialysis, with a 1‐minute interval and the results averaged. Intradialytic BP and heart rate were measured automatically at 30, 60, 120, 180, and 240 minutes by the dialysis apparatus.

Patients were dialyzed on either Monday‐Wednesday‐Friday or Tuesday‐Thursday‐Saturday schedules. Prescriptions for patient's dry weight and antihypertensive drug were made by the nephrologist during their weekly visits. Dry weight was assessed by cardiopulmonary radiology and clinical symptoms including peripheral edema, pulmonary congestion, intra‐ and extra‐dialytic BP and muscle spasm. Excess predialysis weight was defined as the difference between predialysis and dry weight.

#### 2.2.1 BPV and other measurements

We defined patients’ baseline BP as the mean of all predialysis BPs between August 1, 2018, and July 31, 2019. For each BPV measurements, the SD was calculated (SD_SBP_ and SD_DBP_) with the coefficient of variation (CV, CV_SBP_, and CV_DBP_) and the variability independent of the mean (VIM, VIM_SBP_, and VIM_DBP_). The CV was SD factored by mean BP values (M_SBP_ and M_DBP_), and the VIM by the SD factored by the mean to the power x, which was obtained by fitting a curve to the plot of SD against the mean BP level.^14^

The following demographic and clinical data were collected: age, gender, comorbidity (diabetes, hypertension), body mass index (BMI), use of antihypertensive medications, and dialysis vintage. Antihypertensive medications were classified as one of five mutually exclusive classes: β‐blocker–containing regimen (without an RAS agent), RAS‐containing regimen without a β‐blocker (RAS), regimen containing both a β‐blocker and an RAS agent (BB + RAS), and any other antihypertensive regimen without β‐blocker or RAS agent (other). The following laboratory parameters were also collected: Kt/V, blood hemoglobin, serum albumin, calcium, phosphate, and parathyroid hormone (PTH). All laboratory values were measured using standardized automated methods. Laboratory values were measured monthly except PTH that was measured quarterly. The averaged or median values during the exposure period served as the baseline data.

### 2.3 Outcomes

The primary outcome was all‐cause mortality. The second outcome was MACE, a composite of fatal cardiovascular event, nonfatal myocardial infarction, nonfatal stroke, ventricular arrhythmias and limb amputation because of peripheral vascular disease.^6^, ^15^ The ventricular arrhythmias included ventricular tachycardia, ventricular fibrillation, and II‐ or III‐degree atrioventricular block.^16^ Patients were censored at the time of an event of interest occurred.

### 2.4 Statistical analysis

The baseline characteristics of all the patients were compared tertiles of BPV to assess factors that were associated independently with BPV at baseline using linear mixed effects models with a random intercept for the clinic to account for clustering of outcomes by providers. The follow factors were also incorporated as explanatory variables: demographic characteristics (age, sex), clinical factors (history of diabetes, hypertension, smoke, and BMI), heart rate measured simultaneously with blood pressure, dialysis‐related factors (dialysis vintage, Kt/V, and the ratio of excess weight at hemodialysis start to dry weight), laboratory measurements (serum albumin, calcium, phosphate, hemoglobin, and PTH), and use of antihypertensive medications. The association of BPV with outcomes was assessed by discrete time proportional hazards models using binary regression. Hazard ratios were calculated for each outcome per 1 SD increase in BPV after adjustment for the same a pre–defined potential confounders. BPV was categorized into tertiles, and the association with all‐cause mortality was measured using Kaplan‐Meier curves. A sensitivity analyses for BPV was undertaken by tertiles to quantify the association of BPV with outcomes after stratification by categories of SBP at baseline (tertiles of SBP). Statistical significance was taken as *p* < .05 using two‐tailed tests. Statistical analyses were undertaken by using SPSS Statics, version 22.0 (IBM, New York). Kaplan‐Meier curves analysis was performed using R programming language, version 4.1.0.

## 3 RESULTS

### 3.1 BPV metrics

The mean VIM_SBP_ that was used for the BPV metric was 15.52±4.19, and mean VIM_DBP_ was 8.61±2.15 (Table 1).

**TABLE 1:** Blood pressure variability parameters

Parameters	Mean ± SD
**Systolic Blood Pressure**	
1. Mean	138.78±17.29
2. SD	15.49±4.28
3. CV	0.11±0.03
4. VIM	15.52±4.19
**Diastolic Blood Pressure**	
1. Mean	77.75±9.92
2. SD	8.60±2.19
3. CV	0.11±0.03
4. VIM	8.61±2.15

Abbreviations: SD, standard deviation; CV, coefficient of variation; VIM, variability independent of the mean.

### 3.2 Baseline characteristics

**FIGURE 1:**
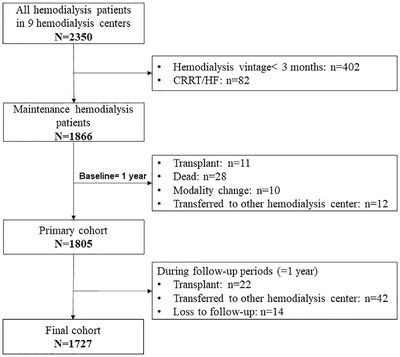
Overview of cohort formation. Selection of the final cohort of 1727 maintenance hemodialysis patients from nine hemodialysis centers in Zhejiang Province of China

The final study cohort comprised 1727 in‐center maintenance hemodialysis patients at nine hemodialysis centers that represented 73% of the initial cohort (Figure 1). Demographic, clinical, and biochemical characteristics are shown in Table 2. Mean age was 59±14 years, and 57% were male, and 11% had a history of smoke. Glomerulonephritis accounted for 56% of the causes of end‐stage renal disease (ESRD), with 75% having a history of hypertension and 24% having diabetes mellitus. The median dialysis vintage was 52 months, and the ratio of excess predialysis weight at start of hemodialysis to dry weight was 4.46±2.46 %.

**TABLE 2:** Baseline characteristics of study population by tertiles of predialysis VIM_SBP_

		Tertiles of VIM_SBP_			
Characteristics	Overall	Lowest (< 13.433)	Middle (13.433‐16.832)	Highest (> 16.832)	*p*
**N (%)**	1727	576	576	575	
**Age (y)**	58.95±13.64	57.24±13.93	59.34±13.22	60.26±13.60	.001
**Male (%)**	983 (56.92)	343 (59.55)	332 (57.64)	308 (53.57)	.112
**Comorbidities**					
Diabetes (%)	416 (24.09)	114 (19.79)	140 (24.31)	162 (28.17)	.004
Hypertension (%)	1289 (74.64)	456 (79.17)	433 (75.17)	400 (69.57)	.001
**BMI (kg/m^2^)**	21.65±4.03	21.59±3.71	21.70±4.33	21.66±4.02	.911
**Smoke (%)**	186 (10.77)	67 (11.63)	62 (10.76)	57 (9.91)	.643
**Cause of ESRD (%)**					.026
Diabetic nephropathy	367 (21.25)	98 (17.01)	124 (21.53)	145 (25.22)	
Hypertensive nephropathy	136 (7.87)	42 (7.29)	44 (7.64)	50 (8.70)	
Glomerulonephritis	964 (55.82)	349 (60.59)	319 (55.38)	296 (51.48)	
Other diagnoses	260 (15.06)	87 (15.10)	89 (15.45)	84 (14.61)	
**Heart rate (per min)**	75.20±7.87	75.10±7.63	75.07±7.75	75.42±8.23	.706
**Dialysis vintage (mo)**	51.63±43.71	46.88±38.08	52.37±46.73	55.66±45.45	.003
**Excess weight at HD start/ Dry weight (%)**	4.46±2.46	4.50±2.40	4.41±2.73	4.45±2.23	.825
**Laboratory parameters**					
Kt/V	1.50±0.64	1.52±0.75	1.49±0.41	1.50±0.71	.631
HB (g/L)	103.60±12.72	104.36±13.04	103.49±11.85	102.95±12.13	.165
Alb (g/L)	39.25±3.45	39.66±3.34	39.33±3.50	38.75±3.45	<.001
Ca (mmol/L)	2.21±0.35	2.23±0.41	2.20±0.41	2.20±0.18	.200
P (mmol/L)	1.69±0.44	1.69±0.41	1.70±0.44	1.69±0.48	.835
PTH (ng/L)	293.60 (157.90‐510.40)	285.99 (149.50‐473.50)	295.86 (168.00‐523.19)	298.13 (155.05‐554.00)	.147
**Use of antihypertensive medications (%)**					.018
Any RAS regimen (without β‐blocker)	304 (17.60)	78 (13.54)	114 (19.79)	112 (19.48)	
Any β‐blocker regimen (without RAS)	272 (15.75)	95 (16.49)	85 (14.76)	92 (16.00)	
β‐blocker + RAS combination	181 (10.48)	54 (9.38)	56 (9.72)	71 (12.35)	
Other medications and combinations	970 (56.17)	349 (60.59)	321 (55.73)	300 (52.17)	

Data are presented as mean (SD) or column percent.

Abbreviations: BMI, body mass index; ESRD, end‐stage renal disease; HD, hemodialysis; HB, hemoglobin; Alb, albumin; PTH, parathyroid hormone; RAS, renin angiotensin system.

### 3.3 BPV and all‐cause mortality

**FIGURE 2:**
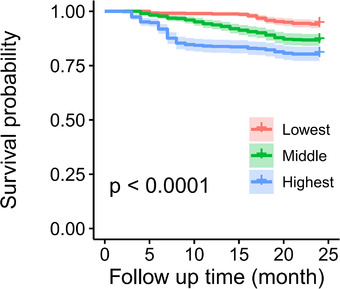
Kaplan‐Meier survival functions for all‐cause mortality according to tertiles of predialysis systolic BPV

In total, 224 deaths occurred in 1727 patients during the follow‐up periods. The risk of all‐cause mortality was associated with higher VIM_SBP_ in both the unadjusted and fully adjusted models (Table 3, Figure 2). In the fully adjusted model, each 1 SD increase in the VIM_SBP_ was associated with 10.1% higher risk of all‐cause mortality (95% confidence interval [95% CI], 6.4% to 12.5%).

**TABLE 3:** Association of predialysis VIM_SBP_ and outcomes in hemodialysis patients

		Crude^a^		Fully Adjusted^b^	
Outcomes	Events	HR (95% CI)	*p*	HR (95% CI)	*p*
**All‐cause mortality**	224	1.110 (1.075‐1.146)	<.001	1.101 (1.064‐1.140)	<.001
**MACE**	299	1.100 (1.069‐1.133)	<.001	1.091 (1.059‐1.125)	<.001

Abbreviations: MACE, major adverse cardiovascular events; HR, hazard ratio; CI, confidence interval.

^a^ Unadjusted model.

^b^ Adjusted for demographic characteristics (age, sex), clinical factors (history of diabetes, hypertension, smoke, and BMI), heart rate, dialysis‐related factors (cause of ESRD, dialysis vintage, Kt/V, and the ratio of excess weight at HD start to dry weight), laboratory measurements (serum albumin, calcium, phosphate, hemoglobin, and PTH), and use of antihypertensive medications.

### 3.4 BPV and MACE

In total, 299 MACEs occurred in 1727 patients during the follow‐up periods. The risk of MACE was associated with higher VIM_SBP_ in both the unadjusted and fully adjusted models (Table 3). In the fully adjusted model, each 1 SD increase in the VIM_SBP_ was associated with 9.1% higher risk of MACE (95% CI, 5.9% to 12.5%).

### 3.5 Sensitivity analyses

After stratification by baseline SBP, the magnitude and direction of the association between VIM_SBP_ and outcomes remained similar to the primary analysis in each of the categories, suggesting that the association between VIM_SBP_ and outcomes was not dependent on baseline BP (Table 4).

**TABLE 4:** Association of predialysis VIM_SBP_ with outcomes after stratification by predialysis SBP categories

Outcomes	BP Range	Events	Crude^a^		Fully Adjusted^b^	
			HR (95% CI)	*p*	HR (95% CI)	*p*
**All‐cause mortality**						
T1	<132	65	1.083 (1.027‐1.142)	.003	1.080 (1.018‐1.146)	.011
T2	132‐146	76	1.119 (1.056‐1.187)	<.001	1.097 (1.028‐1.170)	.005
T3	>146	83	1.140 (1.077‐1.207)	<.001	1.152 (1.080‐1.229)	<.001
**MACE**						
T1	<132	85	1.082 (1.031‐1.136)	.001	1.081 (1.026‐1.139)	.003
T2	132‐146	88	1.107 (1.047‐1.170)	<.001	1.079 (1.017‐1.144)	.011
T3	>146	126	1.119 (1.065‐1.175)	<.001	1.119 (1.061‐1.179)	<.001

HR per 1 SD increase in the predialysis VIM_SBP_. T1 to T3 refers to categories of SBP at baseline (Tertiles of SBP).

Abbreviations: MACE, major adverse cardiovascular events; HR, hazard ratio; CI, confidence interval.

^a^ Unadjusted model.

^b^ Adjusted for demographic characteristics (age, sex), clinical factors (history of diabetes, hypertension, smoke, and BMI), heart rate, dialysis‐related factors (cause of ESRD, dialysis vintage, Kt/V, and the ratio of excess weight at HD start to dry weight), laboratory measurements (serum albumin, calcium, phosphate, hemoglobin, and PTH), and use of antihypertensive medications.

### 3.6 Additional analyses

Higher predialysis VIM_DBP_ was associated with an increased risk of all‐cause mortality in both unadjusted and fully adjusted models. In the fully adjusted model, each 1 SD increase in the VIM_DBP_ was associated with 12.0% higher risk of all‐cause mortality (95% CI, 4.7% to 19.8%) and with 9.1% higher risk of MACE (95% CI, 2.9% to 15.6%) (Table 5).

**TABLE 5:** Association of predialysis VIM_DBP_ and outcomes in hemodialysis patients

Outcomes	Events	Crude^a^		Fully Adjusted^b^	
		HR (95% CI)	*p*	HR (95% CI)	*p*
**All‐cause mortality**	224	1.121 (1.054‐1.192)	<.001	1.120 (1.047‐1.198)	.001
**MACE**	299	1.097 (1.038‐1.160)	.001	1.091 (1.029‐1.156)	.003

Abbreviations: MACE, major adverse cardiovascular events; HR, hazard ratio; CI, confidence interval.

^a^ Unadjusted model.

^b^ Adjusted for demographic characteristics (age, sex), clinical factors (history of diabetes, hypertension, smoke, and BMI), heart rate, dialysis‐related factors (cause of ESRD, dialysis vintage, Kt/V, and the ratio of excess weight at HD start to dry weight), laboratory measurements (serum albumin, calcium, phosphate, hemoglobin, and PTH), and use of antihypertensive medications.

## 4 DISCUSSION

Predialysis SBP is most commonly selected to diagnose and manage BP in hemodialysis patients.^6^ SBP is the major determinant of pulse pressure, since both dependent quite strongly on inelasticity of major conduit vessels and pulse wave velocity. In our study, predialysis VIM_SBP_ was selected to assess the association between predialysis BPV and prognosis in hemodialysis patients. SD, CV and VIM were recognized as the most common BPV metrics.^1^, ^13^, ^17^, ^18^ Since SD and CV are often strongly correlated with mean BP, they displayed minimal discriminatory capacity for BP fluctuations and ambient BP levels. Compared with SD and CV, the VIM is highly independent of the mean BP. In this cohort of 1727 hemodialysis patients, higher predialysis systolic BPV was independently associated with an increased risk of all‐cause mortality and MACE. This association persisted across all baseline SBP categories and after adjustment for a number of potential confounding factors. Thus, the predialysis systolic BPV emerges as a potentially modifiable risk factor for all‐cause mortality and MACE in hemodialysis patients. Greater predialysis diastolic BPV was also found to be independently associated with an increased risk of all‐cause mortality and MACE.

In non‐hemodialysis CKD patients, higher long‐term BPV was found to be associated with cardiovascular events and was considered as a risk factor for all‐cause and cardiovascular mortality.^19^, ^20^, ^21^, ^22^ In hemodialysis patients, few studies have been reported on the association between BPV and cardiovascular outcomes. Most of previous studies assessed BPV over 3 or 6 months, which did not consider seasonal changes in BP.^6^, ^23^, ^24^ In a previous retrospective study, Wang and associates investigated predialysis systolic BPV over 1 year period in 99 hemodialysis patients and found that greater predialysis systolic BPV was associated with long‐term mortality.^13^ In our study, we measured a consecutive 12 months of predialysis blood pressure to determine BPV and found that higher predialysis BPV was significantly associated with the risk of all‐cause mortality and MACE. Thus, strategies to reduce fluctuations in predialysis BP might be considered to improve the long‐term prognosis in hemodialysis patients.

The BPV is one compelling putative risk factor to explain the strikingly high rate of CVD in hemodialysis patients.^25^ Indeed, BPV is significantly associated with CVD and mortality in these patients.^1^, ^4^ Their BPV may have unique determinant including volume and osmolar shifts, and impaired counter‐regulatory responses.^26^ Additionally, BPV in hemodialysis patients may entail vascular remodeling, overactivation of the sympathetic nervous system and loss of compliance of conduit vessels with increases in arterial pulse wave velocity.^27^ These physiologic factors also impair the maintenance of consistent end‐organ perfusion in the event of rapid downward and upward fluctuations in BP, thus exposing patients to periods of tissue hypoxia and capillary shear stress alternation that may lead to further organ damage.^4^ Our data extend the retrospective report on 11291 incident hemodialysis patients treated at 210 dialysis clinics in the United States that greater predialysis systolic BPV was a risk factor for all‐cause mortality, cardiovascular mortality, and cardiovascular events.^6^ A recent retrospective analysis also confirmed above findings.^13^ The BPV metric used in this study was independent of mean BP.^14^ The association between predialysis systolic BPV and outcomes remained significant after stratification by baseline SBP category. Therefore, a goal of management of hemodialysis patients to contest their very high mortality and MACE might be to maintain BP stability.

In the present study, we found that dry weight, or the ratio of excess weight at hemodialysis start to dry weight, was not significantly associated with predialysis BPV. Meanwhile, the adjustment for the dry weight and the ratio of excess weight at hemodialysis start to dry weight in the final outcomes model did not change the association between BPV and outcomes. In a previous retrospective study on the association between predialysis BPV and outcomes, they observed that achievement of prescribed dry weight was associated with lower predialysis BPV, but the adjustment for achievement of prescribed dry weight in the finally outcomes model did not change the association between BPV and outcomes.^6^ Meanwhile, a randomized clinical trial confirmed that dry weight reduction did not affect BPV levels.^28^ It is hypothesized volume losses that occur with dialysis induce baroreceptor‐dependent changed in autonomic function that stabilizes venous return (capacitance vessel constriction) and peripheral constriction (arteriolar constriction) subjects with inelastic conduit vessels cannot adequately sense the fluctuations in BP and do not mount an effective ‐counter‐regulatory response.^27^ BPV reduction is not always accompanied by BP reduction; whether BPV changes depends largely on modification of the responsible pathogenesis, which may be influenced by specific but not all antihypertensive interventions. In our cohort, antihypertensive medications were classified based on the use of β‐blocker–containing regimen and RAS‐containing regimen. The use of antihypertensive medications types at baseline was significantly associated with predialysis BPV, however, the adjustment for antihypertensive medications types in the final outcomes model did not change the association between BPV and prognosis. Previous study found that patients on a non–β‐blocker and non‐RAS antihypertensive regimen had lower BPV compared with those on β‐blocker–containing regimens.^6^ In our study, the proportion of other antihypertensive regimen without β‐blocker and RAS agent was high, which might lead to the fact that the drug‐class did not affect the association between BPV and prognosis. Therefore, further study is needed to investigate interventional methods to improve BPV, and interventional study, if possible, is also needed to explore effects of those interventional methods on outcomes, in hemodialysis patients.

This study aimed to minimize the confounding effect on the measured variables. It is well known that BP fluctuates with season changes.^29^ The aim of our study was to elucidate the relationship between baseline BPV and prognosis during non‐dialysis in hemodialysis patients. In this study, we measured a consecutive 12 months of predialysis BP to determine baseline BPV. The home BP is difficult to standard, and the BP during and after dialysis is more susceptible to the influence of dialysis process.^30^ Therefore, the BP before dialysis can better reflect the baseline blood pressure during non‐dialysis. Predialysis BP was measured when patients took a rest after arriving in waiting room prior to dialysis, which was equivalent to visit‐to‐visit BP.^31^ In this multi‐center prospective observational study, we demonstrated that greater predialysis BPV was associated with all‐cause mortality and MACE. Thus, both reduction in average BP levels and reduction of fluctuations in BP should be emphasized in hemodialysis patients. Interventions aimed at reducing BPV might be beneficial in improving adverse outcomes for hemodialysis patients.

There are several limitations to our study. First, we only explored the association between predialysis systolic and diastolic BPV, but not other components of BP, such as mean arterial pressure, intradialytic or postdialysis BP. Fluctuation in these BP components may be risk factors for poor outcomes and needs additional research. Second, the final cohort recruited most prevalent hemodialysis patients, not incident hemodialysis patients, whose dialysis history might influence the adverse outcome. However, after adjusted for dialysis vintage, the association still remains. Third, this is a prospective observational study. The results can only explain the association and provide clues for treatment of reducing BPV. Further study will be deserved to explore optimal BP management strategy and relevant mechanisms in hemodialysis patients.

In summary, greater predialysis BPV was associated with all‐cause mortality and MACE in hemodialysis patients. Reducing BPV might be beneficial in improving adverse outcomes for hemodialysis patients.

## CONFLICTS OF INTEREST

The authors report no relationships that could be construed as a conflict of interest

## AUTHOR CONTRIBUTIONS

JJ Yang and J Huang performed data interpretation, statistical analysis, and manuscript writing. BY Yu, Q Zhang, SS Zhang, LL Wu, L Luo, LZ Li, and L Li performed data collection. F Han and EY Lai involved in manuscript revision, and supervised the study. Y Yang performed study design and manuscript writing. All authors approved the final version of the manuscript.
